# 203. Evaluating the Impact of Extended-Infusion Beta-Lactam Therapy on Clinical Outcomes and the Subsequent Emergence of Resistance in Adults with Gram-Negative Bloodstream Infections

**DOI:** 10.1093/ofid/ofad500.276

**Published:** 2023-11-27

**Authors:** Sara M Karaba, Jae Hyoung Lee, Suiyini Fiawoo, Sara E Cosgrove, Pranita Tamma

**Affiliations:** Johns Hopkins University, Baltimore, Maryland; Johns Hopkins, Baltimore, Maryland; Johns Hopkins School of Medicine, Baltimore, Maryland; Johns Hopkins School of Medicine, Baltimore, Maryland; Johns Hopkins School of Medicine, Baltimore, Maryland

## Abstract

**Background:**

Data are conflicting regarding the impact of extended-infusion beta-lactam (EI-BL) therapy on patient outcomes. We investigated the impact of EI-BL therapy on clinical outcomes and subsequent emergence of resistance in adults with Gram-negative bloodstream infections (GN-BSI) admitted to 24 US hospitals in 2019.

**Methods:**

Patients who received ≥ 3 days of EI-BL (i.e., ≥ 3-hour infusion) were compared to patients who received the same agents as ≤ 1-hour standard infusions (SI-BL). The EI-BL group underwent 3:1 nearest neighbor propensity score matching without replacement with the SI-BL group based on: Pitt bacteremia score ≥ 4, Charlson comorbidity index ≥ 5, severe immunocompromise, ICU status, source control, active empiric therapy, urinary source, and the 3 most common species. Multivariable regression was applied to the matched cohort to investigate mortality, recurrent infection with the same species, subsequent emergence of resistance (i.e., ≥ 4-fold increase in MIC of the β-lactam used to treat the index BSI), and treatment-related adverse events (AEs), all censored at day 90.

**Results:**

There were 352 and 4,509 patients in the EI-BL and SI-BL groups, respectively. Excellent balance was achieved across all variables in the 3:1 matched cohort. The odds of mortality at day 90 were lower in the EI-BL vs SI-BL group (aOR=0.71, 95% CI 0.52-0.97, p=0.033). When stratified to evaluate the mortality benefit in those with and without severe illness or elevated MICs, a significant mortality benefit was only identified in patients with severe illness and/or elevated MICs. No differences were observed in recurrent infection. There was an increased odds of catheter complications (aOR=3.14, 95% 1.66-5.96, p< 0.001) and early antibiotic discontinuation because of AEs (aOR=3.66, 95% CI 1.68-7.95, p=0.001) in the EI-BL group. Emergence of resistance was similar in the EI-BL and SI-BL groups at 2.9% vs 7.2%, respectively (p=0.351).

**Conclusion:**

The benefits of EI-BL therapy need to be balanced with associated AEs. EI-BL therapy should be prioritized in the severely ill or those infected with non-susceptible organisms. The subsequent emergence of resistance warrants investigation in a larger cohort.

**Disclosures:**

**Sara M. Karaba, MD, PhD, MHS**, Entasis: Advisor/Consultant **Sara E. Cosgrove, MD, MS**, Debiopharm: Advisor/Consultant|Duke Clinical Research Institute: Advisor/Consultant

